# Determination of Amino Acids of Novel Food in Food by HPLC Coupled with Pre-Column Derivatization

**DOI:** 10.3390/foods13244012

**Published:** 2024-12-12

**Authors:** Cenjun Xiao, Jing Xiao, Yuan Wu, Jie Pang, Fuhong Chen, Wenhua Zhang, Dunming Xu

**Affiliations:** 1 Technical Center of Xiamen Customs, Xiamen 361013, China; xiaocenjun@163.com (C.X.); xm3732650@customs.gov.cn (Y.W.); chenfuhong1122@126.com (F.C.); 2 College of Food Science, Fujian Agriculture and Forestry University, Fuzhou 350002, China; pang3721941@163.com; 3 National Center for Food Safety Risk Assessment, Beijing 100022, China; xiaojing@cfsa.net.cn; 4 Technical Center of Hangzhou Customs, Hangzhou 310016, China

**Keywords:** novel food, Theanine, GABA, DABS-Cl, pre-column derivatization, HPLC

## Abstract

A method for simultaneous determination of Theanine and γ-aminobutyric acid (GABA), as the novel food of amino acids, which was established using pre-column derivatization and high-performance liquid chromatography (HPLC). 4-Dimethylaminoazobe nzene-4′-sulfonyl chloride (DABS-Cl) is employed as the derivatization reagent with chromophore linked to Theanine and GABA, which lacks chromophore for DAD analysis in its pristine structure. After the detection wavelength was confirmed, the chromatographic and derivatization conditions were also optimized, including the chromatographic column, mobile phases and their gradient, derivatization temperature and time, the additive amount of buffer solution and derivatization reagent. Methodological verification showed that the derivant of Theanine (DABS-Theanine) and derivant of GABA (DABS-GABA) have good linearity in the range of 1–100 μg/mL, with correlation coefficients of 0.9996 and 0.9995, respectively. The recoveries for both amino acids were between 93.95% and 103.90%, with RSDs ranging from 0.99% to 3.93%. The limit of detection (LOD) was 0.6 mg/kg for Theanine and 0.2 mg/kg for GABA. The limit of quantification (LOQ) was 1.7 mg/kg for Theanine and 0.6 mg/kg for GABA. Furthermore, five commercial products containing Theanine or GABA in two matrices (candy and beverage) were analyzed by the proposed method for validation. The average contents of Theanine and GABA were in the range of 307.49–1312.13 mg/kg and 22.98–6744.55 mg/kg, respectively. The developed method features easy sample pretreatment, a simple derivatization system, good separation specificity, good repeatability, accuracy and reliability, and can meet the large-scale determination Theanine and GABA in various food substrates.

## 1. Introduction

In recent years, novel food continues to develop and gradually come to the public’s attention. As of August 2024, 161 novel foods have been publicized and permitted to be used by the National Health Commission in China. There are various types of novel foods available, among which there are two kinds of novel foods of amino acids, including Theanine and γ-aminobutyric acid (GABA). Theanine is a special amino acid in tea, which is the main component of tea that produces fluid and moistening sweetness. It can improve human immunity and memory ability, act as an anti-depressant, reduce blood pressure, eliminate fatigue, and affect the expression of neurotransmitters in the brain [1,2,3,4,5,6,7]. GABA is an important inhibitory neurotransmitter in the central nervous system of mammals, with various physiological activities such as improving sleep, enhancing liver and kidney functions, assisting in lowering blood pressure and blood sugar, as well as acting as an anti-anxiety and anti-tumor agent [8,9,10,11,12]. In the food market, Theanine and GABA are commonly added to sweets and beverages for daily stress relief and to aid with sleep. With the emphasis on “eating for health”, this functional ingredient is being added more and more to our daily diets. Measures that can conveniently and efficiently improve sleep problems are chosen by more people, and foods that add Theanine and GABA to the market are welcomed by young people, who often suffer with insomnia. Therefore, in order to test whether Theanine and GABA are contained or added to foods, and to check whether the product is in line with the advertised amount of addition, it is very important to establish a simple and quick test method for Theanine and GABA.

Theanine can be detected using near-infrared spectrum instrument technology combined with stoichiometric methods. However, near-infrared spectrum instrument technology is susceptible to interference from other factors, and its spectral data requires preprocessing. The modeling methods are complex, making them unsuitable for routine quantitative detection [13]. Theanine can also be analyzed by high-performance liquid chromatography (HPLC), but chromatographic peaks often overlap due to its weak retention properties and the lack of significant separation between Theanine and other hydrophilic side chain amino acids such as GABA. Therefore, the chromatographic separation of Theanine directly on the instrument is poor and easily disturbed; therefore, it is not suitable for daily detection [14]. Gao et al. extracted green tea samples with acetonitrile, purified them using PSA and C18 QuEChERS methods, and performed qualitative and quantitative analysis of Theanine using gas chromatographytandem (triple quadrupole) mass spectrometry (GC-MS/MS) with multi-reaction monitoring (MRM) mode [15]. Duan et al. determined Theanine in Wuyi rock tea using ultra high-performance liquid chromatography-tandem mass spectrometry [16]. Yi et al. used formaldehyde and sodium borohydride as the main derivative reagents to conduct pre-column derivations of Theanine, and then determined it by capillary electrophoresis with electrochemiluminescence (CE-ECL) [17]. Wang et al. established an effective method for the determination of 20 free amino acids in tea samples using 4-(carbazole-9-yl)-benzyl chloroformate (CBBC-Cl) as a derivatization reagent by high-performance liquid chromatography combined with fluorescence detection (HPLC-FLD) [18]. The derivant of these amino acids, including Theanine, were identified by high-performance liquid chromatography-electrospray ionization-tandem mass spectrometry (LC-ESI-MS/MS). For the detection of GABA, Lu et al. established a colorimetric method based on the reaction of GABA with hypochlorite and phenol in an alkaline solution to produce a blue–green substance. This method involves measuring the absorbance of the colored substance for quantitative analysis of GABA. However, this method is susceptible to interference from other components in the sample and is not suitable for complex samples [19]. Liu et al. developed a method for detecting GABA in alcoholic beverages using liquid chromatography-tandem mass spectrometry (LC-MS/MS) with deuterated isotope GABA-d6 as the internal standard [20]. Cheng et al. established a quantitative method for the determination of GABA in germinated brown rice using o-phthalaldehyde as a derivatization reagent [21]. Zhuang et al. derivated GABA with 9-fluorenylmethyloxycarbonyl chloride (FMOC-Cl) and determined GABA content in fermented soybean products by HPLC. There are various methods for the detection of Theanine and GABA [22].

After comparison, it is found that both of them can be derivated and then determined by HPLC. Tu et al. used o-phthalaldehyde and n-acetyl-l-cysteine as derivatization reagents to establish an HPLC method for the determination of Theanine and GABA in tea [23]. These two non-protein amino acids are difficult to detect using UV detectors due to the lack of ultraviolet absorption functional groups. However, derivatization can introduce the chromophore to the two amino acids, allowing these amino acids to be detected by a UV detector. Commonly used derivatization reagents include dansyl chloride [24,25], 4-dimethylaminoazobenzene-4′-sulfonyl chloride (DABS-Cl) [26,27], o-phthalaldehyde (OPA) [28,29], phenyl isothiocyanate (PITC) [30,31], 2, 4-dinitrofluorobenzene (FDNB) [32,33]. The derivant of OPA and GABA is unstable and begins to decompose after half an hour, making it unsuitable for analyzing large quantities of samples. The derivatization of PITC must be performed under strictly anhydrous conditions, which presents challenges for the experimental environment and practical operation. The derivatization reagent FDNB can interfere with the sample and is a highly toxic substance with strong carcinogenicity; therefore, it is generally not used. The derivatization time of dansyl chloride is long, and the price is high. DABS-Cl reacts with active hydrogen on the primary or secondary amine of amino acids under alkaline conditions, and it removes one molecule of HCl to produce molecules with stronger and more stable fluorescence characteristics, which can solve the phenomenon of non-fluorescence and ultraviolet chromophobe of amino acids. The derivant of DABS-Cl is very stable and sensitive, easy to operate, and reasonable in derivatization time and reagent price, making it an ideal pre-column derivatization reagent. Syu et al. also tried to use the DABS-Cl reagent to derivate amino acids in tea, including Theanine and GABA [34]. In this study, DABS-Cl was used to derivate Theanine and GABA, and a simple, fast, and low-cost method for the detection of Theanine and GABA in food samples by pre-column derivatization combined with HPLC-DAD was established and validated for the first time. This method can be applied to the daily sampling inspections of testing institutions, and it can meet the detection requirements of large quantities of food samples.

## 2. Materials and Methods

### 2.1. Reagents and Chemicals

Theanine (purity ≥ 98.7%) and GABA (purity ≥ 99.6%) were obtained from Alta Scientific Co., Ltd. (Tianjin, China). 4-Dimethylaminoazobenzene-4′-sulfonyl chloride (DABS-Cl) and sodium bicarbonate (NaHCO_3_) were purchased from Macklin Biochemical Technology Co., Ltd. (Shanghai, China). Sodium acetate (CH_3_COONa, HPLC grade) and N,N-dimethylformamide (DMF, HPLC grade) were obtained from ANPEL Experimental Technology Co., Ltd. (Shanghai, China). Acetonitrile (HPLC grade) was obtained from Merck KGaA (Darmstadt, Germany). Acetic acid was purchased from National Pharmaceutical Group Chemical Reagent Co., Ltd. (Shanghai, China). Ultra-pure water was prepared in the laboratory.

### 2.2. Instrumentation and Materials

A high-performance liquid chromatograph UFLCXR and photodiode array UV-Visible light detector SPD-M20A 230V (Shimadzu, Kyoto, Japan) were used for detection analysis. The electronic balance JY5002 (Sainy Hengping Scientific Instrument Co., Ltd., Shanghai, China) and XS105DU (METTLER TOLEDO, Zurich, Switzerland) were used for weighing samples and standards. The sample was mixed with a multi-tube vortex mixer MS200 (Ruicheng Instrument Co., Ltd., Hangzhou, China). The ultrasonic cleaner S22 (ZEALWAY, Wilmington, DE, USA) was used to extract samples. The pH meter FE20-FiveEasy (METTLER TOLEDO, Zurich, Switzerland) was used to regulate the pH of the mobile phase. The PTFE syringe filter (Dikma Technology Co., Ltd., Beijing, China) was used to filter the derivant. These three columns, Platisil ODS column (4.6 × 250 mm, 5 μm) (Dikma Technology Co., Ltd., Beijing, China), Kinetex C18 100Å column (4.6 × 250 mm, 5 μm) (Phenomenex, Torrance, CA, USA) and Capcell Pak ADME-HR column (4.6 mm × 250 mm, 5 μm) (OSAKA SODA, Osaka, Japan) were used to compare the separation results and select the final column to use.

### 2.3. Preparation of Solution

Theanine standard stock solution: 50 mg Theanine (accurate to 0.01 mg) was dissolved in ultra-pure water, with a constant volume of 50 mL, that is, 1000 μg/mL standard reserve solution, and stored in the refrigerator at 4 °C for later use. GABA standard stock solution: 50 mg GABA (accurate to 0.01 mg) was dissolved in ultra-pure water, with a constant volume of 50 mL, that is, 1000 μg/mL standard reserve liquid, and stored in the refrigerator at 4 °C for later use. Mixed standard intermediate solution: Theanine standard stock solution, GABA standard stock solution, and ultra-pure water were evenly mixed in a ratio of 1:1:8 to obtain 100 μg/mL of the mixed standard working solution, which was stored in the refrigerator at 4 °C. Series mixed standard working solution: the mixed standard stock solution was accurately absorbed and diluted step by step with ultra-pure water to obtain the mixed standard working solution with mass concentrations of 1, 5, 10, 20, 50 and 100 μg/mL, which were stored in the refrigerator at 4 °C for later derivatization. NaHCO_3_ buffer: 4.00 g NaHCO_3_ was dissolved in ultra-pure water, with a constant volume of 100 mL. DABS-Cl derivatization reagent: 0.20 g DABS-Cl powder was dissolved with acetonitrile, with a constant volume of 100 mL, and stored in the refrigerator at 4 °C for later use. CH_3_COONa mobile phase: 2.05 g CH_3_COONa was dissolved in ultra-pure water, with a constant volume of 1000 mL, 4%DMF was added, and the pH was adjusted to 6.5 with acetic acid. The mobile phase was filtered with a 0.45 μm water phase filter membrane, and then ultrasound was performed for 5–10 min.

### 2.4. Preparation and Extraction of Sample

The solid food samples were evenly mixed, thoroughly crushed into fine particles with a grinder, homogenized and put into sealed sample bags. Dry products were stored at room temperature, and wet products were frozen below zero. The liquid food was homogenized, put into a sealed sample bottle, and kept refrigerated at 4 °C. Food samples are from random sampling of the market conducted by customs units.

Then, 1.00 g sample was accurately weighed into a 50 mL centrifuge tube, and 25 mL ultra-pure water was added and swirled for 10 min. Ultrasonic extraction was conducted at room temperature for 20 min, and centrifuged at 8000 r/min for 3 min. The supernatant was transferred to a 50 mL volumeter bottle, the sample residue was extracted with 20 mL ultra-pure water according to the above steps, the two supernatants were combined, and the constant volume was adjusted with ultra-pure water to 50 mL. It was then shaken well to obtain the sample solution and set aside for derivatization.

### 2.5. Pre-Column Derivatization

A sample solution of 1 mL was accurately measured, 50 μL of NaHCO_3_ buffer and 1 mL of DABS-CI derivatization reagent were added, and the mixture was mixed and reacted in a constant temperature water bath at 70 °C for 20 min. The mixture was removed, cooled to room temperature, and then it was filtered through a 0.22 μm membrane before HPLC analysis.

### 2.6. Chromatographic Conditions

Capcell Pak ADME-HR column (5 μm, 4.6 mm × 250 mm) was used as the analytical column. The flow rate was 1.0 mL/min. The injection volume was maintained at 10 μL. The wavelength used was 463 nm. The column temperature was set at 30 °C, and elution was performed in gradient mode using 25 mmol/L of sodium acetate solution (containing 4% DMF) (A) at a pH of 6.5 and acetonitrile (B) as the mobile phase. The composition of the mobile phase varied as follows: 0–9.00 min, isocratic condition, 32% B; 9.00–15.00 min, increased to 44% B; 15.00–17.00 min, isocratic condition, 44% B; 17.00–21.00 min, decreased to 32% B; 21.00–24.00 min, isocratic condition, 32% B.

### 2.7. Optimization of Chromatographic Conditions

Using 25 mmol/L sodium acetate (4%DMF, pH = 6.5)-acetonitrile (68:32, *v*/*v*) as a mobile phase, the separation effects of Platisil ODS column, Kinetex C18 100Å column and Capcell Pak ADME-HR column for derivant were compared. The column with the best separation effect was selected as the final column used in this study.

After selecting the best column, the ratio of the mobile phase was adjusted to make the peak time reasonable, the peak and peak type narrow and sharp, and the chromatographic system stable. Finally, the suitable chromatographic conditions were obtained.

### 2.8. Optimization of Derivatization Conditions

By derivating 100 μg/mL mixed standard intermediate solution under different conditions, using the response peak area as the index, the effects of different derivatization temperature (30, 40, 50, 60, 70, 80, 90, and 100 °C), derivatization time (10, 20, 30, 40, 50, 60, 70, 80, and 90 min), the additive amount of buffer solution (0, 50, 100, 150, 200, 250, 300, 350, and 400 μL) and the additive amount of derivatization reagent (0.6, 0.7, 0.8, 0.9, 1.0, 1.1, 1.2, 1.3, and 1.4 mL) on the derivant were investigated, and the optimal derivatization system was obtained.

## 3. Results and Discussion

### 3.1. Classification of Chromatographic Peaks and Confirmation of Detection Wavelength

**Figure 1:**
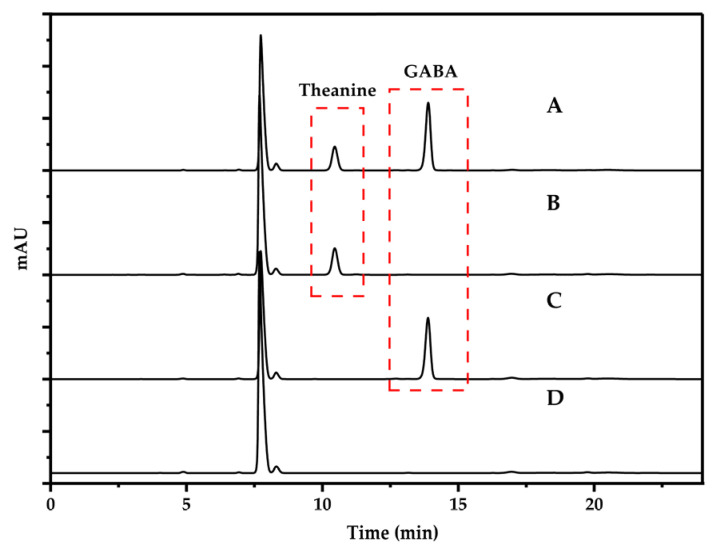
Chromatograms of solution after DABS-Cl derivatization: mixed standard intermediate solution (**A**), Theanine standard solution (**B**), GABA standard solution (**C**) and ultra-pure water (**D**).

The 100 μg/mL mixed standard intermediate solution, Theanine standard solution, GABA standard solution and ultra-pure water were, respectively, derived according to Step 2.5, and the derived products were detected (Figure 1).

**Figure 2:**
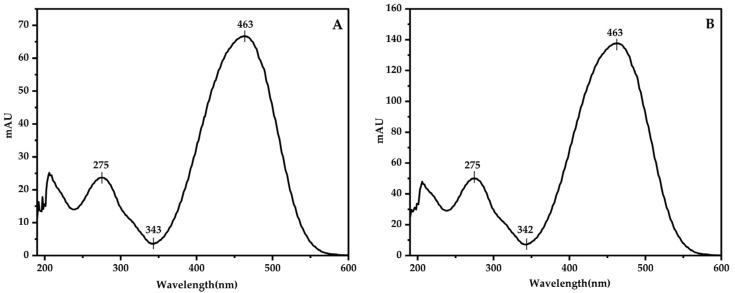
The spectrum of the standard solution of DABA-Theanine (**A**) and DABA-GABA (**B**).

The experiment investigates the impact of detection wavelengths on the sensitivity of detecting the target compound, Theanine, and GABA. Using a DAD, a full scan of the target compound was conducted within the wavelength range of 210–600 nm. The absorption spectra obtained are shown in the figure below. Both the DABS-Theanine and DABS-GABA exhibited a maximum absorption peak at a wavelength of 463 nm. Therefore, 463 nm was chosen as the detection wavelength in this experiment (Figure 2).

### 3.2. Selection of Chromatographic Column

**Figure 3:**
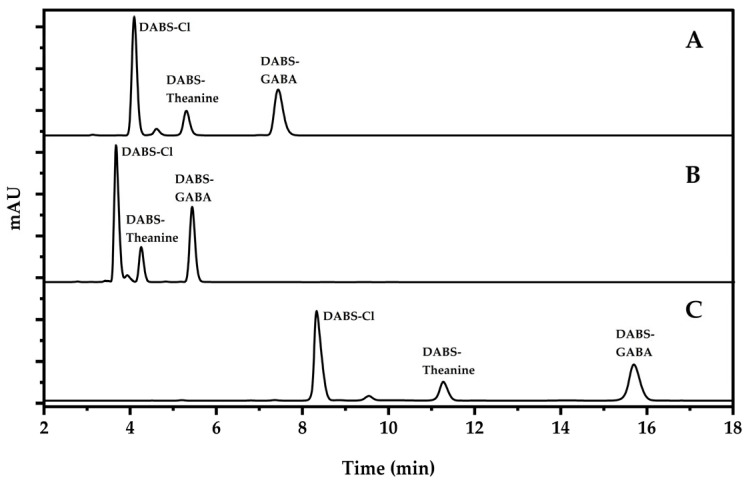
Chromatogram of separation effect of different chromatographic columns. (A: Platisil ODS column; B: Kinetex C18 100Å column; C: Capcell Pak ADME-HR column).

The chromatographic separation of Theanine and GABA is poor and easily disturbed in the daily detection. To solve this problem and improve chromatographic separation, the following chromatographic columns of different materials and types were compared in the experiment: Platisil ODS column (4.6 × 250 mm, 5 μm), Kinetex C18 100Å column (4.6 × 250 mm, 5 μm), and Capcell Pak ADME-HR column (4.6 mm × 250 mm, 5 μm). The results showed that the separation of Theanine and derivatization reagent is insufficient on the Platisil ODS column and the Kinetex C18 100Å column, and the retention time of Theanine was close to the retention time of the derivatization reagent (Figure 3A,B). The separation effect of the derivatization reagent, Theanine, and GABA was relatively good on the Capcell Pak ADME-HR column (Figure 3C). Therefore, the Capcell Pak ADME-HR column was selected the separation column.

### 3.3. Optimization of Gradient Elution Conditions

**Figure 4:**
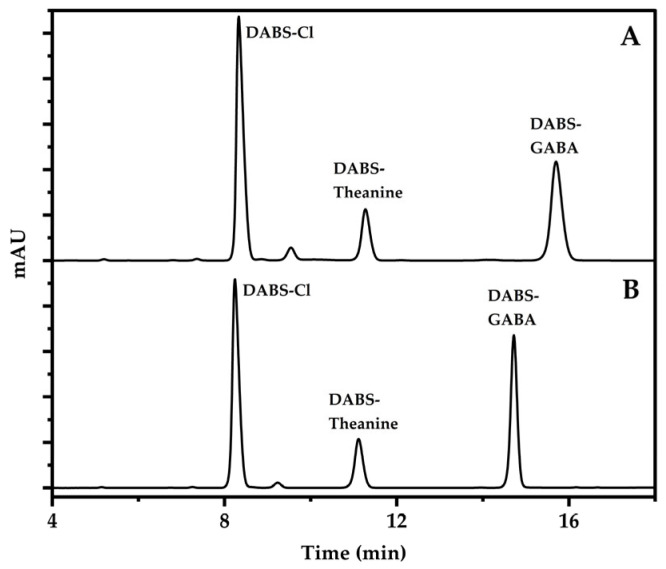
Chromatograms of different mobile phase gradients. (A: Isocratic elution; B: Gradient elution).

DMF is a polar solvent that is often added to the mobile phase in liquid chromatography to increase the polarity of the mobile phase, making the components of the sample with similar polarity more easily separated [35]. When the mobile phase was a phosphate-buffer solution—methanol, the isocratic elution ability was weak, the elution time was long, and the baseline was unstable. The elution time was shortened and the baseline was stable when sodium acetate solution-acetonitrile was selected as the mobile phase [16]. When 25 mmol/L sodium acetate (4%DMF, pH = 6.5)-acetonitrile (68:32, *v*/*v*) was used to eluate the derivative reagent, the results indicated that the peak shape of the target compound (DABS-GABA) was not sharp enough. The ratio of the mobile phase was adjusted so that the derivatization reagent peaked first, and then the ratio of acetonitrile was increased, resulting in increased elution strength, narrow and sharp chromatographic peaks of Theanine and GABA, and other impurities that can flow out of the column faster, reducing the impact on the next sample injection, and shortening the entire chromatographic analysis time (Figure 4).

### 3.4. Optimization of Derivatization Conditions

#### 3.4.1. Derivatization Temperature

**Figure 5:**
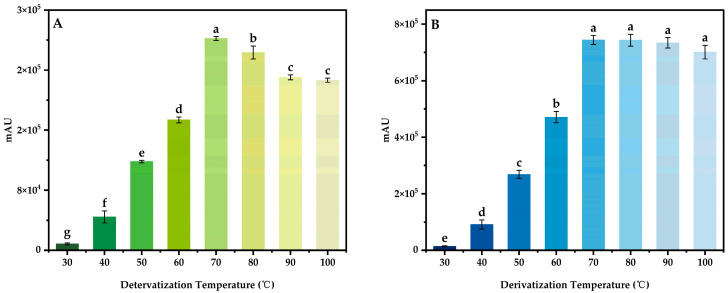
Effect of derivatization temperature on peak area of DABS-Theanine (**A**) and DABS-GABA (**B**). Bars with different letters indicate significant differences (*p* < 0.05).

The derivatization time was set to 20 min, the additive amount of buffer and the derivatization reagent were set to 200 μL and 0.4 mL, respectively. The mixed standard intermediate solution of 100 μg/mL was derivated at different temperatures, three parallel tests were carried out for each variable, and the results were shown in the figure below (Figure 5), the results of significant analysis are marked with letters. The results indicated that the peak areas of DABS-Theanine and DABS-GABA increase step by step with the increase in derivatization temperature, and there is a significant difference. When the derivatization temperature reaches 70 °C, the peak area of DABs-Theanine and DABS-GABA reaches the maximum. As the temperature continues to rise, part of the derivatization reagent is decomposed, and the binding of the derivatization reagent with Theanine and GABA reaches saturation and no longer increases significantly. At the same time, there was also partial decomposition of the derivant caused by the high temperature, especially DABS-Theanine. Therefore, the optimal derivatization temperature was 70 °C.

#### 3.4.2. Derivatization Time

**Figure 6:**
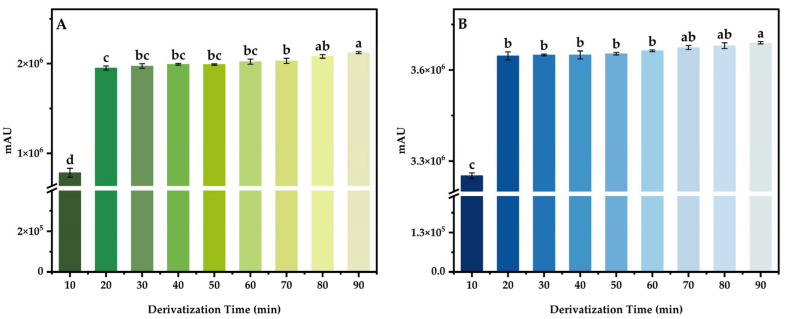
Effect of derivatization time on the peak area of DABS-Theanine (**A**) and DABS-GABA (**B**). Bars with different letters indicate significant differences (*p* < 0.05).

The derivatization temperature was set to 70 °C, the additive amount of buffer and derivatization reagent were set to 200 μL and 0.4 mL, respectively. The mixed standard intermediate solution of 100 μg/mL was derivated at different times, and three parallel tests were conducted for each variable. The results are shown in the figure below (Figure 6). The results show that the peak area of DABS-Theanine and DABS-GABA increased with the extension of derivatization time. In the first 20 min, DABS-Cl was efficiently combined with Theanine and GABA, and the peak area was greatly increased. However, when the derivatization time exceeded 20 min, the Theanine and GABA in solution had basically formed a stable derivant with DABS-Cl, and the peak area of DABS-Theanine and DABS-GABA could only produce quite small gradient changes. Considering the actual experimental conditions, it is meaningless to prolong the derivatization time, so the optimal derivatization time was 20 min.

#### 3.4.3. The Additive Amount of Buffer Solution

**Figure 7:**
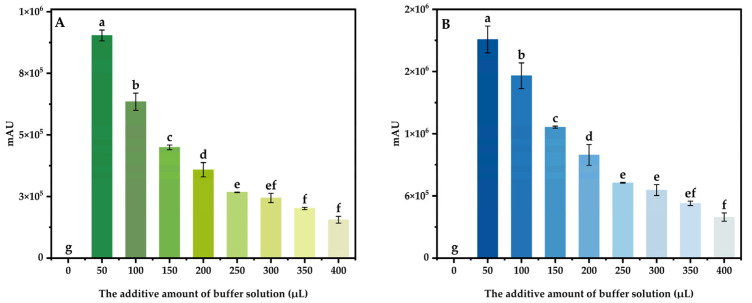
Effect of the additive amount of buffer solution on the peak area of DABS-Theanine (**A**) and DABS-GABA (**B**). Bars with different letters indicate significant differences (*p* < 0.05).

The derivatization temperature and time were set to 70 °C and 20 min, respectively, and the additive amount of the derivatization reagent was set to 0.4 mL. Adding different amounts of buffer solution to the mixed standard intermediate solution of 100 μg/mL, three parallel tests were conducted for each variable, and the results are shown in the figure below (Figure 7). The results show that the derivatization of DABS-Cl needed to be carried out under alkaline conditions, and the sodium bicarbonate solution is weakly alkaline. Without adding sodium bicarbonate solution to the derivatization system, the reaction was not able to proceed. The peak area of DABS-Theanine and DABS-GABA reached the maximum when the buffer solution was added to 50 μL. When the addition amount exceeded 50 μL, the content of sodium bicarbonate increased with the additive amount of buffer solution, resulting in the pH of the derivatization system increasing, and the binding bond of DABS-Cl with Theanine and GABA being destroyed, which affected the derivatization reaction and led to a sharp decline in the peak area of DABS-Theanine and DABS-GABA. Therefore, the optimal additive amount of buffer solution was 50 μL.

#### 3.4.4. The Additive Amount of Derivatization Reagent

**Figure 8:**
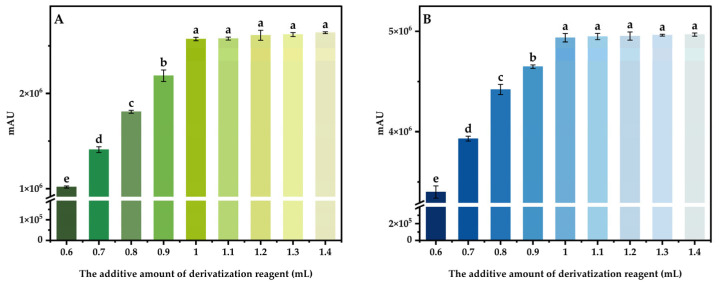
Effect of the additive amount of derivatization reagent on the peak area of DABS-Theanine (**A**) and DABS-GABA (**B**). Bars with different letters indicate significant differences (*p* < 0.05).

The derivatization temperature and time were set to 70 °C and 20 min, respectively, and the additive amount of buffer solution was set to 50 μL. Adding different amounts of derivatization reagent to the mixed standard intermediate solution of 100 μg/mL, three parallel tests were carried out for each variable. The results are shown in the figure below (Figure 8). The results indicated that DABS-Theanine and DABS-GABA were continuously produced and changed significantly with the increasing amount of derivatization reagents. When the amount of derivatization reagent added exceeded 1.0 mL, the mass concentration of Theanine and GABA in the solution was limited, and the binding with DABS-Cl reached a peak, and no more Theanine and GABA were combined with the excess DABS-Cl. The whole derivatization system showed a saturated state, and the gradient of the peak area of DABS-Theanine and DABS-GABA slowed down and tended to be stable. Therefore, in order to save reagents and reduce unnecessary waste, the optimal additive amount of derivatization reagent was 1.0 mL.

### 3.5. Method Validation

#### 3.5.1. Linearity, LOD, and LOQ

**Figure 9:**
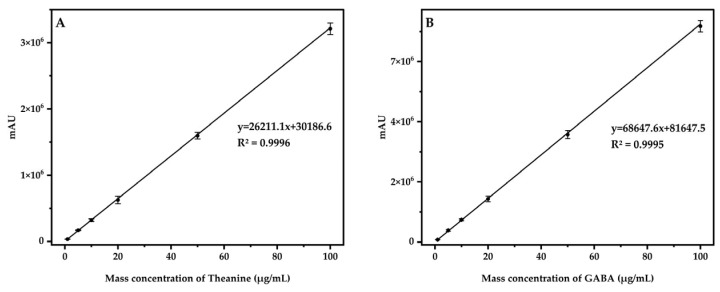
Standard curve of Theanine (**A**) and GABA (**B**).

The series mixed standard working solution in Section 2.5 was derived and determined according to chromatographic conditions. Following the operating parameters of HPLC-DAD, the standard curve was plotted with the mass concentration on the horizontal coordinate and the corresponding peak area value on the vertical coordinate (Figure 9). The experiments showed a satisfactory linear relationship within the range of 1 to 100 μg/mL for Theanine and GABA standard solution, with correlation coefficients (R^2^) all greater than 0.9995. The limit of detection (LOD) was calculated from the signal-to-noise ratios (LOQ = 3 × S/N). The LODs were 0.6 mg/kg (Theanine) and 0.2 mg/kg (GABA). The limit of quantitation (LOQ) was calculated from the signal-to-noise ratios (LOQ = 10 × S/N). The LOQs were 1.7 mg/kg (Theanine) and 0.6 mg/kg (GABA).

#### 3.5.2. Precision and Stability Test

The derived mixed standard working solution of 50 μg/mL was continuously injected six times to measure the peak area and to calculate the relative standard deviation (RSD). The results are shown in the table below (Table 1). The RSD of the peak area of DABS-Theanine is 0.08%, and that of DABS-GABA is 0.10%, indicating that the method has good precision and high accuracy.

The derived mixed standard working solution of 50 μg/mL was injected at 0, 2, 4, 6, 8, 12, and 24 h, respectively, to measure the peak area and calculate the RSD. The results are shown in the table below. The RSD of peak area of DABS-Theanine was 0.37%, and that of DABS-GABA was 0.49%, indicating that the method had good stability at 24 h and could be applied to the treatment of large quantities of samples (Table 2).

#### 3.5.3. Recovery and Repeatability

Recovery experiments were conducted on different samples with concentrations of 50, 100, and 200 mg/kg, representing low, medium, and high concentrations, respectively. Theanine and GABA are often added to candies and beverages, so the recovery rates of these two food substrates were measured. At the same time, the recovery rates of carbohydrate and protein substrates were also determined. The recovery rates ranged from 93.95% to 103.90%, with RSDs below 3.93% (Table 3). These recovery rates and precision values comply with the requirements of the current European guidelines (SANTE/11312/2021) (recovery in the range 70–120%, and RSD ≤ 20%, retention time ± 0.1 min) [36], meeting the analysis requirements for Theanine and GABA in food. This method can be used for routine analysis and detection.

### 3.6. Method Application

**Figure 10:**
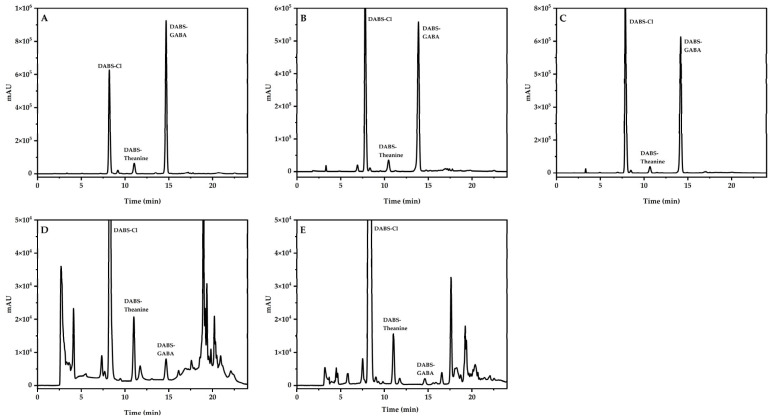
Chromatograms of actual samples. ((**A**) Theanine and GABA bonbon; (**B**) Theanine and GABA gummy candy; (**C**) Theanine and GABA mixed drink; (**D**) Black tea beverage; (**E**) Solid beverage).

In today’s food market, it is often seen that some candies and beverages have added Theanine and GABA, and the words “Add novel food” and “Theanine and GABA can relieve stress and improve sleep” can be seen on their packaging and promotional pages to attract customers. Tea drinks are also marketed as being rich in Theanine. Theanine and GABA were quantitatively detected in five different kinds of samples, and the chromatogram of each sample was shown in the figure below (Figure 10). Six parallel tests were conducted for each sample (Table 4). Two candies on the market were tested, one of which was Bonbon, a form in which the outer layer of sugar is wrapped around the inner layer, which makes it easier to add Theanine and GABA to the product. However, this also leads to less uniform sample preparation and greater detection errors. Three types of beverages were also tested: a functional drink, a tea soft drink, and a solid beverage of tea by-products. The average content of Theanine was 307.49–1312.13 mg/kg, and the average content of GABA was 22.98–6744.55 mg/kg. According to the recommended intake amounts in the products, these five products meet the requirement that the daily intake of Theanine does not exceed 400 mg/day and that GABA does not exceed 500 mg/day, as stated in the regulations [37,38].

### 3.7. Comparison with Other Methods

To evaluate the performance of this method, the key indicators including LOD, LOQ, linear range, derivative reagent, derivative time, separation time, and RSD of the method were compared with other published literature (Table 5). Compared with other methods, this method involves a wider range of food substrates and types. Because of its advantages of stability, simplicity, and quickness, as well as its compatibility with common laboratory liquid phase instruments, this method can be widely used in inspection institutions. In the long-time test of large-scale samples, it can still maintain a good response. The chromatographic analysis time is short, and the content of Theanine and GABA in the sample can be quickly determined. In particular, compared to the study conducted by Syu et al. [34], this method is faster and more accurate. Syu et al. used DABS-Cl reagent to derivate and detect a variety of amino acids in tea, which resulted in a very long chromatographic analysis time. Under the chromatographic conditions selected by Syu et al., the peak time of GABA was even close to 40 min. In this study, Theanine and GABA could be detected in about 15 min by selecting a suitable chromatographic column and changing the mobile phase gradient. Syu et al. focused on detecting and comparing the contents of Theanine and GABA in different tea leaves, while this study focused on optimizing the derivatization conditions and establishing a method capable of detecting Theanine and GABA in food through strict methodological verification, and this method could better adapt to daily laboratory detection.

## 4. Conclusions

In this study, DABS-Cl reagent was used for a pre-column derivatization HPLC method to simultaneously determine the content of Theanine and GABA—two amino acids in novel food. The separation effect of different chromatographic columns was investigated, and the mobile phase proportional gradient was optimized. By setting the single factor experiment of four factors, including derivatization temperature, derivatization time, the additive amount of buffer and derivatization reagent, the derivatization conditions were optimized. The optimal derivatization conditions were as follows: 50 μL of sodium bicarbonate buffer and 1 mL of DABS-Cl derivatization reagent were added to 1 mL of sample solution and were derived in a water bath at 70 °C for 20 min. Under the condition of gradient combination of sodium acetate and acetonitrile as a mobile phase, the derivant was separated by a Capcell Pak ADME-HR column and detected by a DAD detector. According to the established method, Theanine and GABA showed a good linear relationship in the range of 1–100 μg/mL, with R^2^ was 0.9996 and 0.9995, respectively. The recoveries were in the range of 93.95–103.90%, and the RSD was controlled in the range of 0.99–3.93%. The LODs of Theanine and GABA were 0.6 mg/kg and 0.2 mg/kg, respectively. The LOQs of Theanine and GABA were 1.7 mg/kg and 0.6 mg/kg, respectively. The method is characterized by easy sample pretreatment, a simple derivatization system, a short derivatization time, and good precision and reproducibility. The derivant remains stable for 24 h, making this method suitable for the large-scale determination of Theanine and GABA in various food substrates. In addition, the method will provide some analytical methods for the subsequent review and detection of amino acids in novel food.

## Figures and Tables

**Table 1:** The results of six consecutive injections of 50 μg/mL mixed standard working solution.

Serial Number	The Peak Area of DABS-Theanine (mAU)	RSD (%)	The Peak Area of DABS-GABA (mAU)	RSD (%)
1	1,447,947	0.08	3,695,706	0.10
2	1,449,504		3,694,736	
3	1,446,784		3,685,472	
4	1,447,279		3,693,983	
5	1,449,319		3,691,117	
6	1,447,828		3,694,924	

**Table 3:** Recoveries and RSDs of the target analytes spiked in different sample matrices (*n* = 6).

Sample	Additive Amount (mg/kg)		Recovery (%)						RSD (%)
Crisps	50	Theanine	95.18	96.68	102.18	99.08	99.18	100.28	2.54
		GABA	101.97	99.77	95.77	98.27	101.87	100.97	2.41
	100	Theanine	97.39	97.69	96.24	103.19	98.34	100.34	2.54
		GABA	98.13	100.78	101.13	98.08	99.88	102.13	1.65
	200	Theanine	101.12	99.05	100.35	99.67	100.62	96.10	1.82
		GABA	102.54	101.72	102.07	103.24	99.67	98.57	1.78
Coated Tofu	50	Theanine	97.70	100.40	101.70	95.60	98.60	101.10	2.34
		GABA	100.50	97.90	99.10	98.50	101.60	98.60	1.41
	100	Theanine	97.40	96.10	103.65	102.25	101.70	99.70	2.94
		GABA	97.50	99.80	94.40	103.90	102.65	103.05	3.70
	200	Theanine	101.45	95.23	98.15	101.15	102.18	93.95	3.53
		GABA	102.85	101.50	97.33	102.13	99.90	100.95	1.95
GABA Candy	50	Theanine	99.20	94.30	97.90	103.00	96.60	103.70	3.70
		GABA	103.87	100.47	98.17	99.87	102.67	99.47	2.11
	100	Theanine	98.85	98.75	98.55	97.95	100.90	97.10	1.28
		GABA	94.53	99.63	101.33	94.93	98.83	100.63	2.96
	200	Theanine	102.43	97.60	99.25	97.93	98.43	98.75	1.76
		GABA	94.59	100.29	96.14	103.37	94.82	95.87	3.62
Green Tea Beverage	50	Theanine	99.70	95.30	102.40	99.50	103.40	99.20	2.84
		GABA	101.60	96.10	98.50	96.90	103.80	98.80	2.93
	100	Theanine	99.90	98.30	99.35	99.00	96.90	94.40	2.08
		GABA	98.40	98.65	97.90	102.40	97.45	98.75	1.79
	200	Theanine	95.75	95.75	97.70	95.78	95.25	103.15	3.11
		GABA	99.13	98.08	103.58	98.10	100.70	101.45	2.15
Matcha Cookie	50	Theanine	98.98	97.08	97.88	101.68	100.08	98.88	1.64
		GABA	98.82	94.02	98.92	98.42	95.82	96.92	2.01
	100	Theanine	94.09	96.69	99.64	97.64	95.89	99.74	2.26
		GABA	94.76	98.01	97.06	99.46	99.71	96.56	1.92
	200	Theanine	99.27	100.77	99.35	99.75	101.27	98.30	1.08
		GABA	98.30	96.23	99.80	99.88	94.85	98.75	2.07
Solid Beverage	50	Theanine	99.52	98.82	96.22	96.42	102.82	102.22	2.81
		GABA	97.77	96.27	97.57	95.37	99.17	100.97	2.05
	100	Theanine	98.26	101.16	94.81	96.81	96.51	101.51	2.73
		GABA	94.73	99.83	96.53	94.48	94.98	98.03	2.22
	200	Theanine	97.10	100.48	96.73	99.60	97.13	96.85	1.66
		GABA	103.79	100.49	97.27	97.22	95.92	96.89	3.02
Theanine and GABA Mixed drink	50	Theanine	95.97	103.67	98.27	101.57	102.77	102.57	3.00
		GABA	101.08	100.38	99.88	101.38	98.68	99.68	0.99
	100	Theanine	102.83	96.83	101.83	96.58	94.13	103.43	3.93
		GABA	97.74	99.44	94.14	99.64	98.79	102.69	2.83
	200	Theanine	102.67	101.59	95.29	99.99	99.34	97.59	2.70
		GABA	101.15	97.70	98.20	99.47	96.90	98.47	1.51

**Table 4:** The results of actual samples (*n* = 6).

Samples		Content (mg/kg)						Average Value (mg/kg)	RSD (%)
Theanine and GABA bonbon	Theanine	1330.10	1313.75	1336.80	1309.65	1264.05	1318.40	1312.13	1.96
	GABA	7088.40	7012.10	6528.75	6687.05	6563.10	6587.90	6744.55	3.61
Theanine and GABA gummy candy	Theanine	1093.10	1100.20	1118.15	1110.80	1100.90	1130.15	1108.88	1.23
	GABA	5282.20	5134.90	5299.75	5211.30	5129.65	5158.25	5202.68	1.43
Theanine and GABA mixed drink	Theanine	768.75	768.20	774.40	785.20	762.90	773.85	772.22	0.99
	GABA	5778.50	5744.25	5800.25	5817.75	5785.55	5826.35	5792.11	0.51
Black tea beverage	Theanine	375.60	366.85	370.20	371.95	366.80	373.65	370.84	0.97
	GABA	22.90	22.50	22.60	23.30	23.35	23.25	22.98	1.62
Solid beverage	Theanine	302.40	302.55	312.75	305.70	307.55	314.00	307.49	1.62
	GABA	93.50	90.65	91.90	94.35	91.70	91.50	92.27	1.50

**Table 5:** Comparison of the introduced method with other methods.

Analytes	Samples	LOD (mg/kg)	LOQ (mg/kg)	Linear Range (µg/mL)	Derivatization Reagent	Derivatization Time (min)	Separation Time (min)	RSD (%)	Method	Ref.
Theanine	Green tea	3 × 10^−3^	0.01	0.02–1.0	-	-	16	3.0–10.5	GC-MS/MS	Gao et al. 2023 [15]
Theanine	Tea	9.8 × 10^−2^	-	0.87–34.8	Formaldehyde and sodium borohydride	-	5	-	CE-ECL	Yi et al. 2017 [17]
Theanine	Tea	5.7 × 10^−4^	1.88 × 10^−3^	10–200	CBBC-Cl	6	14	2.24–4.85	HPLC-FLD	Wang et al. 2020 [18]
GABA	Alcoholic beverage	0.1–8.2	0.25–27.3	10–500	-	-	6	1.17–7.93	LC-MS/MS	Liu et al. 2023 [20]
GABA	Germinated brown rice	9.8 × 10^−4^	-	5–60	OPA	2	20	-	HPLC-DAD	Cheng et al. 2014 [21]
GABA	Fermented soybean products	3	10	1–100	FMOC-Cl	5	45	0.56–4.21	HPLC-DAD	Zhuang et al. 2023 [22]
GABA	Plants foods and medicinal plants	9	-	2–1000	Dansyl-Cl	60	-	0.73–3.72	HPLC-DAD	Pencheva et al. 2022 [24]
Theanine and GABA	Tea	2.6 and 4.1	8.7 and 13.7	1.74–69.6 and 1.03–41.2	OPA and NAC	1	19 and 18	-	HPLC-FLD	Tu et al. 2012 [23]
Theanine and GABA	Tea	1.162 × 10^−2^ and 3.4 × 10^−3^	-	-	DABS-Cl	10	20 and 40	-	HPLC-DAD	Syu et al. 2008 [34]
Theanine and GABA	Food	0.6 and 0.2	1.7 and 0.6	1–100	DABS-Cl	20	11 and 15	0.99–3.93	HPLC-DAD	This method

**Table 2:** The detection result of 50 μg/mL mixed standard solution within 24 h.

Time (h)	The Peak Area of DABS-Theanine (mAU)	RSD (%)	The Peak Area of DABS-GABS (mAU)	RSD (%)
0	1,447,947	0.37	3,695,706	0.49
2	1,447,828		3,694,924	
4	1,441,699		3,647,233	
6	1,447,330		3,679,871	
8	1,449,575		3,659,602	
12	1,434,174		3,681,925	
24	1,443,504		3,681,633	

## Data Availability

The original contributions presented in the study are included in the article, further inquiries can be directed to the corresponding authors.
